# A blueprint for strengthening climate and health literacy through professional adaptability

**DOI:** 10.3389/fpubh.2023.1112944

**Published:** 2023-03-23

**Authors:** Maggie L. Grabow, Valerie J. Stull, Micah B. Hahn, Vijay S. Limaye

**Affiliations:** ^1^Department of Family Medicine and Community Health, School of Medicine and Public Health, University of Wisconsin-Madison, Madison, WI, United States; ^2^Center for Sustainability and Global Environment, University of Wisconsin-Madison, Madison, WI, United States; ^3^Institute for Circumpolar Health Studies, University of Alaska, Anchorage, Anchorage, AK, United States; ^4^Science Office, Natural Resources Defense Council, New York, NY, United States

**Keywords:** climate change, health education, training, literacy, professional adaptability

## Abstract

Responding effectively to intensifying climate change hazards to protect human health in personal and professional settings is an urgent and pressing challenge. This will require collaboration and subject matter expertise of people across the life course and occupations. In this perspective piece, we build on a previously published compilation of climate and health literacy elements to explore tangible opportunities to strengthen climate and health understanding among individuals spanning educational levels, professional settings, and societal needs. Educational materials addressing climate change and health linkages have historically focused on K-12, college, post-graduate education, and continuing medical education, with less attention devoted to reaching students in trade schools and other professional settings. Here, we outline a flexible blueprint for strengthening climate and health literacy among all people by targeting education in a way that is relevant for each age group or profession. In particular, we discuss the idea of professional adaptability as a way to design practical climate and health training for people currently in the workforce.

## 1. Introduction

Climate change is a human health emergency (1). Numerous climate-sensitive health risks are well-established, including mortality from heatwaves, respiratory ailments from smog ozone and allergenic pollen, mental and physical effects due to wildfires and migration, increased transmission of vector-borne diseases, injuries from flooding, and undernutrition stemming from reduced crop yields (2–4). The urgency to act to reduce greenhouse gas emissions emitted when burning fossil fuels and from large scale deforestation is paramount. The world has warmed ~2°F compared to pre-industrial times, and 18 of the 19 hottest years ever recorded have occurred since 2000 (3).

Despite scientific evidence demonstrating the link between climate change and human health, this relationship is not well-understood by the general public and many professionals. In the U.S., for example, only about half of Americans believe climate change poses a risk to their personal wellbeing (5). While public understanding of the scientific fundamentals of climate change in the US (e.g., that there is scientific consensus that global warming is occurring and is driven by human activities) has improved slightly since 2010, it remains low overall (6). Perceptions of climate change risk among Americans, on the other hand, have increased consistently since 2008, but reflect a view that risk is greater for people and animals living “far away” from them (in space or time) compared to localized, individual, or community risk (5–7).

This lack of understanding of how the climate influences individuals and society and how individuals influence the climate is considered poor *climate change literacy* (also called climate literacy) (8). Climate literacy, as an educational framework, describes essential principles of climate science and overlays them with basic science literacy benchmarks. Climate literacy also defines one's ability to assess credible scientific information, communicate clearly, and make informed decisions related to behaviors that impact the climate. In its sixth assessment report, the Intergovernmental Panel on Climate Change (IPCC) recently emphasized that “Enhancing climate change literacy on impacts and possible solutions is necessary to ensure widespread, sustained implementation of adaptation by state and non-state actors…Ways to enhance climate literacy and foster behavioral change include access to education and information…” (9). Report co-chair Hans-Otto Porner also argued, “We have an education gap and an implementation gap” (10).

**Figure 1:**
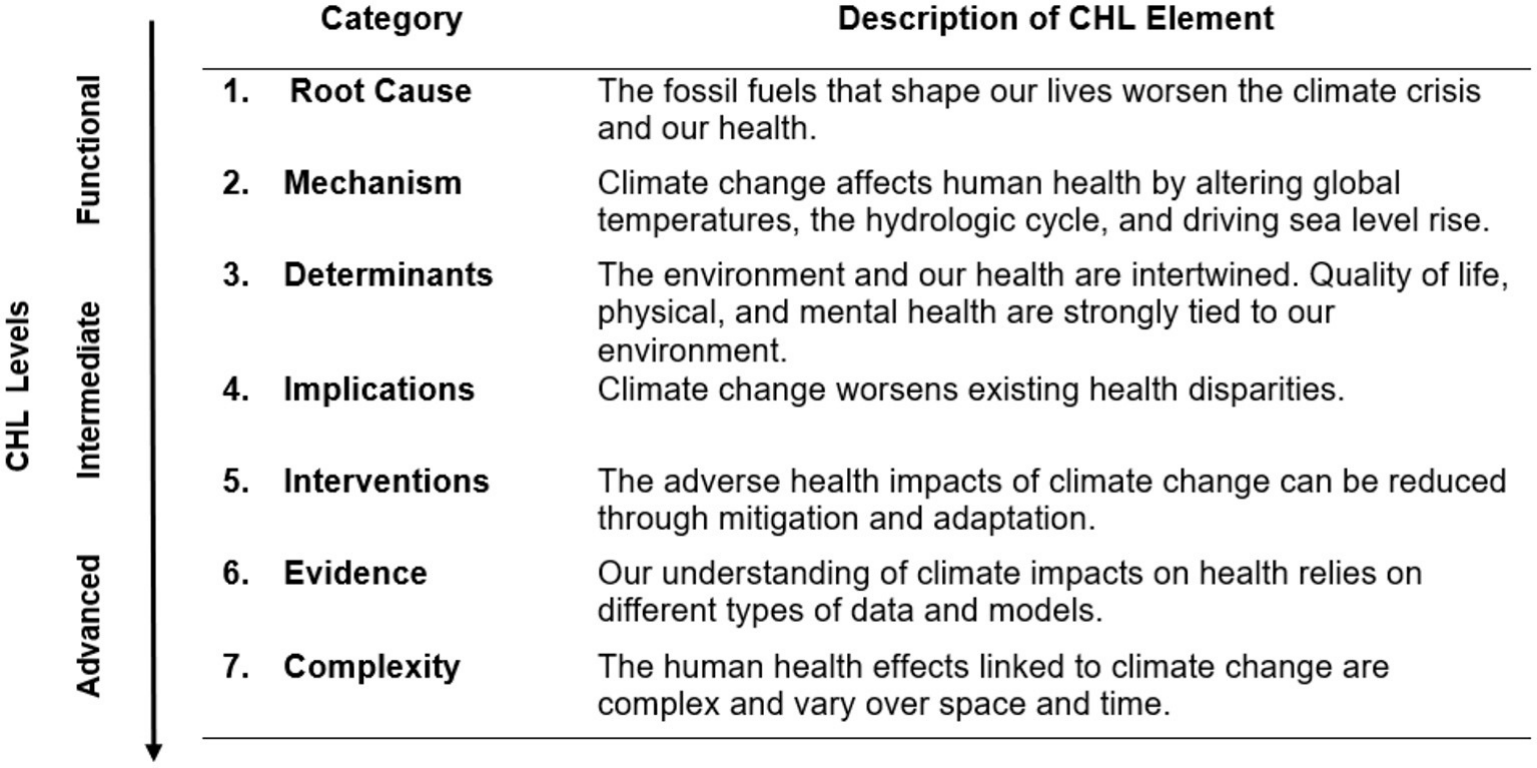
Climate and health literacy (CHL) elements as adapted from Limaye et al. (14).

In the US, current national science curriculum standards do not broadly emphasize climate change content (11), nor the profound health implications of climate change. Current pressures to attain educational standards amidst challenging staff turnover in the wake of COVID-19 present an additional challenge to adequately prepare students for a world increasingly disrupted by a changing climate (12, 13). In a prior analysis of the existing training landscape (14), we identified major health content gaps in climate literacy elements, which are the only federally-endorsed criteria for climate change training in the US. To address this shortcoming, we have previously developed a set of seven *climate and health literacy* (CHL) elements that can help to standardize and strengthen climate change curriculum development by linking climate content to relatable human health concerns (7, 14, 15). These elements are shown in Figure 1 and are categorized into three literacy levels, functional, intermediate, or advanced.

Central to our concept of CHL is the degree to which one understands *complexity* in the relationship between climate change and health *via* both direct and indirect linkages, as well as the ability to make informed decisions based on such knowledge. CHL builds on an incomplete characterization of the health impacts of climate change as included in climate change literacy (8); specifically, it includes understanding of how mitigation and adaptation can reduce harms to physical and mental health (16), the overlap between fossil fuel dependency and worsening health (1), as well as the economic costs associated with climate-sensitive health impacts (14, 17, 18). Not all students or people need to engage in training to advance from functional, to intermediate, to advanced levels of CHL. Younger learners, for example, might begin with functional CHL, learning the root causes of mechanisms of climate change and overlaps with health. Professionals and educators, on the other hand, need intermediate and advanced literacy levels in order to better affect change in their respective fields. The real-world application of CHL requires an educational framework for various ages and learning groups such to help them master content relevant to their daily lives and professions. This strategy should be tailored to the needs of the target population. A one-size fits all approach would be inappropriate.

Responding effectively to intensifying climate change hazards to protect human health through education is an urgent and pressing need (19). Building CHL is essential across the population. The current and future challenges posed by climate change require collective action; individual action is necessary but insufficient (20–22). We believe an effective response will require collaboration, education, and subject matter expertise of people across industries and throughout society. To accomplish this goal, we must establish and advance a foundational understanding of the relationship between climate change and human health across society for all types of learners.

To date, climate change and health education has been focused predominantly within fields of medicine and public health (14). However, a rapidly changing climate necessitates resilience and adaptation among people of all professionals and life stages. To this end, we offer a blueprint for increasing climate and health literacy *via* strategic education efforts targeting individuals from kindergarten to retirement specifically within the United States, with potential for expansion and translation worldwide. Our blueprint builds on previous work defining CHL (14) by offering a practical guide for achieving CHL objectives across specific audiences. While Limaye et al. (14) identified specific learning objectives, the blueprint presented here provides pedagogical and curricular approaches to meet these objectives. A critical piece of this blueprint is the role of *professional adaptability* in framing climate and health education for the working population (23). Climate and health education is essential for offering people in the workforce skills to anticipate and accommodate changes (e.g., technological, competitive) important to one's profession and to allow for the capacity to modify elements of professional practice accordingly due to changes in the climate.

## 2. Approach

### 2.1. Audience

Most efforts to develop and evaluate climate change educational materials have focused on K-12, college, and graduate education (24–26). Collectively, these audiences represent a critical demographic for early education about climate change and continues to play a major role in pushing for aggressive climate action (27). Less attention has been paid to reaching students in trade schools, despite their potential as a key conduit of information to the public. For example, housing contractors and HVAC professionals can convey information about building design to protect inhabitants from locally-relevant climate-sensitive exposures like extreme precipitation or wildfire smoke.

Recent strategies targeting clinical and public health practitioners provide examples for reaching practicing professionals who are already in the workforce and are required to complete professional continuing education to maintain their credentials (28). However, there are a number of other industries where continuing education is not necessarily “part of the job,” requiring new and creative mechanisms for teaching climate and health. For example, public service professionals, policymakers, farmers, and ranchers may not be exposed to formal continuing education courses. And yet, for these individuals, *professional adaptability* is especially crucial in a changing climate and can be a useful foundation for climate and health education. We know that adaptability alone refers to our human capacity to respond to change, uncertainty, and variability (29). However, in the context of a changing climate and the workplace, we suggest professional adaptability training. As described above, we define professional adaptability as the capacity for climate-informed adaptations in professional practice. For example, first responders might receive training on climate hazard scenarios that they may have to respond to in their community. With intensifying and increasing climate hazards, the necessity for on-the-job decision-making is critically important. Moreover, professionals should receive preparation through profession-specific training modules that include the tenets of climate and health literacy.

Finally, comprehensive climate and health education for the whole population will require consideration of people outside the traditional student or working populations, including life-long learners (e.g., retirees) (30). With regard to climate and health literacy, one of the major distinguishing features of this population is the role of life experience in their understanding of climate change. In contrast to current students, most of today's adult learners likely have not been exposed to information about climate change in formal coursework when they were in school and likely can draw on personal and place-based observations when discussing climate change (31).

### 2.2. Teaching approach

A core strategy in building climate and health literacy is adopting a *meeting people where they are* teaching approach. This broad pedagogy has been applied widely to other disciplines and topics, including design (32), equity and justice (33), and in healthcare and public health (34). A person-, human-, or student-centered approach is intended to improve knowledge attainment and sometimes shift behavior, giving emphasis to the personal characteristics and experiences of students, as opposed to traditional “content-centered learning” (35). Student-centered learning emphasizes active, not passive, learning, as well as efforts to build deep understanding, increased responsibility and accountability for the student, autonomy, and mutual respect between teacher and learner (36). Efforts to meet people and students where they are can be physical, in that they require going to where target audiences are located or reside, but they can also be ideological, contextual, and personal. Importantly, we see this approach as a means to explain concepts and ideas such that they are absorbed by representing them in an intriguing manner that resonates with students' lived experience. Previous research has identified focusing on personally relevant and meaningful information and using active and engaging teaching methods as potentially useful teaching strategies for effective environmental education (37). Moreover, other scholars have argued that the gap between educational vision and teaching practice can be overcome by adopting pedagogical approaches that contribute to the “development of a person as a whole” (38).

Given the urgency of the climate crisis, building a cohort of individuals across the population with at least functional climate and health literacy is essential to building resilience. Deep expertise of climate and health (reaching advanced literacy) is not necessary for the average person, nor is it realistic. We propose, rather, that educational efforts aim to help most people reach functional climate and health literacy at a minimum (Figure 1). Students who complete high school (grade 12), for example, should graduate with functional climate and health literacy or higher. If individuals enter the workforce with a foundation of climate and health literacy, we posit that they will be better positioned to embrace and grasp the premise of professional adaptability. Just as a better-informed citizenry can help reduce vulnerabilities and enhance resiliency of ecosystems impacted by climate change *via* better policy and decision making (8), improved climate and health knowledge can improve adaptation and decision-making to protect health. To achieve this goal, efforts to improve climate and health literacy cannot be limited to formal education settings. Below we outline a guide for integrating this education in various settings throughout the life course.

### 2.3. Blueprint for incorporating climate and health literacy across audiences

To elucidate tangible opportunities that strengthen understanding of climate and health among individuals spanning educational levels, professional settings, and societal positions, we offer a blueprint for building both climate and health literacy and professional adaptability by audience (Table 1). This blueprint for climate and health literacy rests on three guiding principles described above: (1) professional adaptability is a crucial skill in today's workforce and has practical appeal to working professionals, (2) climate and health education efforts will be most effective if they utilize student-centered learning approaches, and (3) developing functional climate and health literacy across the population is more important than deep expertise among a few professionals. With these values in mind, this blueprint offers the building blocks for adapting climate and health education to any audience and level of interest in the topic. In lieu of endorsing specific curricula for climate and health education, we provide example activities that can be adapted to the appropriate educational context.

**Table 1:** Blueprint for building climate and health literacy (CHL) and professional adaptability by audience.

**CHL elements**	**Levels 1–2 (functional)**		**Levels 3–5 (intermediate)**	**Levels 6–7 (advanced)**
**Audience**	**Tier 1: Fast pass exposure**	**TIER 2: Breaking the ice (professional development training and enrichment)**	**Tier 3: A la carte learning (for credit options)**	**Tier 4: Apprenticeship (immersive curriculum)**
**Students**				
K-12	• 4 × 15-min videos, with teacher led discussion and activities. Separate video set targeting elementary, middle, and high school students.	• Extracurricular enrichment opportunities: summer camps, field trips, club activities(making climate and health relevant through local examples, hands-on, engaging activities, and practical skills training) • Certification *via* Cooperative Extension 4-H Program	• Not applicable for K-8 • High school elective class	• See available higher education options in local area
Undergraduate and community college programs	• University common book program • Freshman year orientation to climate and health opportunities on campus or in community • Campus events (speakers, film screenings)	• Elective courses and Summer experiential classes • Critical Analysis of news articles	• Certificate/minor linked with degree • Internships (for credit)	• Degree program in climate change health • Undergraduate multi-semester research and honors experiences
Trade School (e.g., plumbing, HVAC, carpentry, electrician)	• 4 × 15 min interactive videos • Profession-specific Fact Sheets • Yearly CHL Refresher Breakfast	• Workplace training/Online trade-specific training • Self-directed learning options • Certification *via* Cooperative Extension Program	• Climate and health certification • Work with consultants to help a business specialize in CHL • Continuing education course or retreat (3–5 days)	• See available higher education options in local area
Graduate and professional programs (non-health)	• Videos, seminars, brief readings/fact sheets	• Conferences or a 1–2 Day workshop • Critical analysis of news articles	• Certificate/Minor linked with degree • 1 credit seminars • Internships (for credit)	• Build into thesis work/professional capstone/Internships • Collaboration with health faculty • Case example: CHANGE at UW-Madison
Clinical and public health graduate and professional programs	• Video as part of orientation process/week + campus events • Included as part of core professional development curriculum	• Conferences or 1–2 Day workshop • Critical analysis of news articles	• Certificate/minor linked with degree • Internships (for credit) • MPH online major in climate and health	• Critical analysis of science studies • Recurring Research seminars with writing requirements • Professional conferences • Case example: Capstone experience
**Practicing professionals and current workforce**				
Current teachers and faculty	• Handouts, journal articles, or videos • 30-60 min interactive videos • Orientation training • Discipline Specific Fact sheets	• Webinar/1-2 day workshop • Team training	• Faculty learning community that meets regularly • See this: https://facultyforafuture.org/	• Recurring Research Seminars with writing requirements • Professional Conferences • Designated ways to help teachers evaluate CHL of their students
Non-health professionals (lawyers, architects, engineers, journalists)	• 4 × 15 min interactive video • Profession-specific fact sheets • Orientation training	• Continuing education 1-day workshop	• Semester-long continuing education • Continuing Education Retreats (2-5 days)	• Train-the-Trainer Model: Achieving designated CHL subject matter expert status for educating Tier 1/Tier 2 level
**Audience**	**Tier 1: Fast pass exposure**	**TIER 2: Breaking the ice (professional development training and enrichment)**	**Tier 3: A la carte learning (for credit options)**	**Tier 4: Apprenticeship (immersive curriculum)**
Clinical and public health practitioners	• Handouts, journal articles, or videos	• Conference session • Webinar for continuing medical education	• Semester-long continuing medical education • Continuing Education Retreats (2-5 days)	• Recurring Research Seminars with writing requirements • Professional Conferences (or sections at conferences)
Public service professionals (e.g., fire fighters, police officers, EMT, military, crossing-guards)	• Job onboarding/training (e.g., how climate hazards will affect their work) • Trade Specific Fact sheets • 4 × 15 min interactive video module • Yearly CHL Refresher Breakfast	• Continuing education (1-day workshop) • Scenario planning and job-specific climate hazard case studies workshop • Certificate *via* Cooperative Extension Program	• Continuing Education Retreats (2-5 days)	• Train-the-Trainer Model: Achieving designated CHL subject matter expert status for educating Tier 1/Tier 2 level
Policymakers (municipal, local, statewide, regional, national)	• 4 x 15 min interactive video modules • Short white papers with corresponding short presentations • Locale Specific Fact sheets Example: https://iclei.org/en/webinars/TR~	• Workshops at policy conferences: https://climateadaptationforum.org/	• Continuing education retreats (2-5 days)	• Train-the-Trainer Model: Achieving designated CHL subject matter expert status for educating Tier 1/Tier 2 level
Farmers, ranchers, and other agricultural managers	• Extension outreach • Continuing education • Conferences for targeted audiences put on by local Universities • 4 × 15 min video modules • Learning Hubs • Yearly CHL refresher breakfast	• Certification *via* local Cooperative Extension program • Training or education on Incentives for taking action on climate change + health and Scenario planning and job-specific climate hazard case studies workshop	• Continuing education retreats (1-3 days)	• Train-the-Trainer Model: Achieving designated CHL subject matter expert status for educating Tier 1/Tier 2 level
**Other interested individuals**				
Retirees, church groups, community organizations, non-profit organizations	• Videos (PBS NOVA type documentary) • Discuss/distribute articles in popular media	• 1 or 2 day workshop hosted by local organization • Certification *via* Cooperative Extension program	• Local academic lectures or view online lectures • Translation of big analyses (e.g., IPCC, National climate assessment, lancet countdown) • Continuing education retreats (3-5 days)	• Train-the-Trainer Model: Achieving designated CHL subject matter expert status for educating Tier 1/Tier 2 level

We focus on three overarching categories of learners: (1) students (spanning K-12, undergraduate and community colleges, trade schools, and graduate programs including health and non-health related fields), (2) practicing professionals in the current formal workforce, and (3) other interested individuals, especially those out of the formal workforce (e.g., retirees). For each audience, we propose four tiers of engagement to build climate and health literacy, categorized in terms of the degree of time, effort, and planning involved in each level. The following sections summarize proposed activities within each tier, including examples of current resources that suit each tier.

We term Tier 1 “Fast Pass Exposure” intended to provide audiences with an introduction to climate and health literacy concepts [discussed more fully in (14)] on a broad level, especially the focus of the first climate and health literacy element on the basic mechanism by which fossil fuel reliance affects climate change and human health. Tier 1 activities for K-12, undergraduate, and trade school students can include short instructional videos and teacher-led discussions on the links between climate change and health. Offerings here could also include broader environmental education field activities to familiarize learners, especially the youngest ones, with the interconnectedness of human and natural systems. Materials to support Tier 1 activities could be developed by emphasizing the health-relevant content of existing videos and training modules centered on building general climate literacy, including those developed by the US National Oceanic and Atmospheric Administration (39), public broadcasters (40), academics (41), and non-governmental organizations (42, 43).

Tier 2 is referred to as “Breaking the Ice,” with activities intended to strengthen professional development training and knowledge enrichment, with an emphasis on the health dimensions of the climate change problem. These activities build out functional climate and health literacy (14) to include information on the direct mechanisms by which climate change affects human health (e.g., altering global temperatures, the global hydrologic cycle, and sea level rise). These activities might include short electives or summer courses, building on existing training opportunities such as climate and health “bootcamps” that feature presentations from health experts that can familiarize participants with climate change content and its relevance to public health (44). While Tier 2 activities would be structured around professional development and knowledge enrichment rather than academic credit, upon completion participants could earn informal designations as trainees and ambassadors, potentially as a “train the trainer” model to expand the reach of Tier 2 content.

Tier 3 and 4 activities are more formal, structured instructional programs that provide students with expanded academic training in climate and health literacy content. Tier 3, or “A la carte learning,” spans intermediate climate and health literacy elements (health determinants, the implications of climate change to worsen existing health disparities, and opportunities to intervene to address health consequences of climate change *via* mitigation and adaptation) (14). This set of activities involves a smaller range of academic credit options, including high school elective classes, University level certificate/minor programs or semester-long internships (offered during the academic year or summer) (45), continuing education classes for a more focused, customizable learning experience. Health professional students across subject matter areas could access Tier 3 content *via* formal professional development, such as the University of Colorado's innovative Diploma in Climate Medicine, which trains students to develop leadership skills on climate and health science policy, workforce training, research, and linkages to environmental justice (46).

Tier 4, is the most advanced and called “Apprenticeship” level training. It includes the most immersive climate and health literacy curriculum intended to enable advanced mastery of climate and health literacy elements, including an appreciation for the far-reaching evidence upon which climate-health understanding is based and continues to expand, and the complex mechanisms by which climate change effects on human health varies over space and time (14). Tier 4 activities could include entire University-level degree climate and health literacy programs and climate concentrations within Masters of Public Health degrees (47), capstone experiences, or recurring research seminars with required writing components. These formal academic training activities could include development of a climate and health certificate or full degree program, respectively. For example, Vanderbilt University recently introduced their new Climate Studies major that could easily provide a foundation for exploring careers in climate and health. This initiative is innovative because rather than isolating the major in the environmental studies department, it is trans-institutional with required courses in natural sciences, social sciences, and the humanities (48). Completing Tier 4 also lends itself to formal accreditation programs, such as the hypothetical Fellow of the Climate and Health Literacy Society or an accredited Climate and Health Professional program. Lastly, we view individuals in Tier 4 who may be outside of academia but have emerged as CHL subject matter experts to be essential in educating those in lower Tiers.

## 3. Discussion

Achieving the coordinated and substantial societal shift required to curb climate change requires motivating behavior change of a large segment of the global population. We argue that increased education and training to build climate and health literacy (essentially a foundational understanding of climate change and how it affects human health), may be an effective way to accomplish this goal. Further, we offer a blueprint for building this instruction into the lifecourse through already existing educational routes. A key tenant of this blueprint is a student-centered approach that focuses on the learners' context. For example, we provide examples for tailoring this education to the participants' age, previous exposure to climate change education, interest level, and/or profession.

Momentum is already building to formalize climate training for students globally. For example, in response to mounting climate change concerns from university students, leaders at the University of Barcelona have planned a mandatory course on climate change for all students beginning in 2024 (49). As such instruction becomes more prevalent in the years to come, it is important that this training focus on equipping students with concrete knowledge about the threats posed by climate change to their health and wellbeing, and promote development of skills and techniques to build resilience amongst learners. And, since these student skills are inherently interdisciplinary, climate and health-literate learners are both marketable and transferable, assets that could lead to more career options in the future.

One of the major strengths of this approach is that it can be implemented at many levels today, without starting from scratch or reinventing the curricular wheel. Some universities already have climate change preparedness and climate change adaptation for emergency management classes in existence [e.g., (50)]. These classes could potentially be added to and modified for existing undergraduate and graduate training, or even amended for associate's degree programs/trade school programs. By integrating health content into existing formal and informal educational training platforms on climate change, implementation of this approach may be less financially costly and time-intensive than establishment of entirely new teaching programs. Additionally, the majority of US states have already have cooperative extension programs in place with missions to assist in preparing for and responding to emergencies, protecting the environment, and empowering people to adapt to changing technologies. Thus, enhancing cooperative extension programming to promote professional climate adaptability may be an effective way to reach people outside of the traditional student population.

A potential limitation of this approach is that for professionals interested in advanced tiers of education, the development of curricula, content delivery, and the coordination of formal continuing education credit must be governed by an official professional body. One example of a potential governing body would be *The American Society of Adaptation Professionals (ASAP)*, which is a professional home for people who are preparing from climate impacts in their jobs, in their communities, and in their fields of practice. The ASAP hosts education and training for climate adaptation and resilience professionals. While this governing body is currently for climate adaptation professionals, perhaps its model could be expanded to fields that are not explicitly focused on climate adaptation, yet are significantly affected by climate change.

Despite scientific consensus that climate change is real, there will continue to be climate change deniers in every occupation at every age group. The purpose of climate and health literacy is not necessarily to convince people that climate change is real. However, one overarching message of this CHL education initiative is to (1) ***convey*** the elevated risk to human health resulting from increasing exposure to climate hazards and (2) ***determine*** what is and ***execute*** the best course of action for resilience and adaptability in reference to specific professions. Moreover, by utilizing the “meeting people where they are” approach, this increases likelihood of assimilation among learners, no matter where they stand in accepting the science behind climate change.

As the changing climate continues to dramatically alter society and endanger public health (1), it is essential that a broad segment of the population is knowledgeable about the causes and impacts of climate change. To catalyze and sustain climate change action and adaptation, we believe targeted education highlighting climate and health literacy should be established across the life course. This blueprint is a foundation for a practical, transformational educational initiative across the US, with great potential to translate and expand worldwide.

## Data availability statement

The original contributions presented in the study are included in the article/supplementary material, further inquiries can be directed to the corresponding author.

## Author contributions

MG is a post-doctoral fellow in the Department of Family Medicine and Community Health in the UW Madison School of Medicine and Public Health, and also co-led this effort with VS, MH, and VL. VS is a Research Scientist at the University of Wisconsin – Madison, Center for Sustainability and the Global Environment. MH is an Associate Professor of Environmental Health at the Institute for Circumpolar Health Studies at the University of Alaska-Anchorage. VL is a Scientist at the Natural Resources Defense Council. All authors participated in the conception of the perspective piece, reviewed relevant research, designed the blueprint, and conducted much of the writing of this manuscript. All authors contributed to the article and approved the submitted version.
